# A systems microbiology framework for reproducible multi-dataset omics integration with application to long COVID

**DOI:** 10.3389/fsysb.2026.1873899

**Published:** 2026-09-03

**Authors:** Brigitta Varga, Marlet Martinez-Archundia, Linette E. M. Willemsen, Johan Garssen, Alejandro Lopez-Rincon

**Affiliations:** 1 Informatics Institute, University of Amsterdam, Amsterdam, Netherlands; 2 Laboratory for the Design and Development of New Drugs and Biotechnological Innovation, Higher School of Medicine, National Polytechnic Institute (IPN), Mexico, Mexico; 3 Division of Pharmacology, Utrecht Institute for Pharmaceutical Sciences, Faculty of Science, University of Utrecht, Utrecht, Netherlands; 4 Global Centre of Excellence Immunology, Danone Nutricia Research, Utrecht, Netherlands

**Keywords:** algorithmic integration, feature selection, gene expression, multi-dataset omics, systems microbiology, machine learning

## Abstract

Integrative systems microbiology increasingly relies on algorithmic approaches capable of extracting biologically meaningful patterns from heterogeneous and often high dimensional, low-sample-size (HDLSS) biological datasets. A major obstacle in this setting is the instability of inferred molecular signatures across cohorts, tissues, and measurement platforms. Here, we address this problem by formulating molecular system inference as a multi-dataset integration task and by applying the Matthews Correlation Coefficient–Recursive Ensemble Feature Selection (MCC-REFS) algorithm to jointly analyze five independent transcriptomic datasets spanning peripheral blood mononuclear cells, whole blood, plasma, and post-mortem tissues. We compared MCC-REFS with three commonly used feature-selection strategies, GRACES, SelectKBest, and Deep Neural Pursuit (DNP), in order to evaluate robustness, convergence, and cross-context reproducibility. MCC-REFS consistently converged on a compact seven-gene system (*PPP2CB*, *SOCS3*, *ARG1*, *IL6R*, *ECHS1*, *FZD2*, *TRGV3/5*) exhibiting higher stability indices and stronger classification performance than alternative methods. Generalization was assessed using an independent multi-layer perceptron classifier across validation cohorts with differing tissue origin and sequencing technologies, demonstrating preservation of discriminative structure. To support interpretation, we integrated functional, pharmacological, and interventional knowledge from *DrugBank*, *DGIdb*, and *Open Targets*, enabling the mapping of inferred gene systems onto pathways, known drug targets, and ongoing clinical investigations. Taken together, this work presents an algorithmic framework for multi-dataset and multi-omics integration in systems microbiology, illustrating how stable and interpretable molecular patterns can be identified from heterogeneous data, with Long COVID serving as a representative case study.

## 1 Introduction

Integrative systems microbiology aims to characterize biological regulation by jointly analyzing molecular, clinical, and functional data across heterogeneous settings. A persistent challenge in this field is the limited reproducibility of inferred molecular signatures across studies, driven by high dimensionality, sample-constrained cohorts, variability in biological compartments, and differences in experimental platforms. As a result, molecular findings that appear robust within a single dataset often fail to generalize, restricting their interpretability and limiting their utility for mechanistic analysis. Here, systems microbiology is used in the broad host–microbe systems sense. Although this study does not directly analyze microbial community composition, Long COVID and COVID severity comes from a post-infectious condition following SARS-CoV-2 exposure, making host transcriptomic responses relevant for studying infection-associated immune and metabolic dysregulation (Moolamalla et al., 2021; Gardinass et al., 2020).

Long COVID, also referred to as post-acute sequelae of SARS-CoV-2 infection (PASC), offers a representative example of this broader systems-level problem. The condition is characterized by symptoms that persist for weeks or months following acute infection and involve multiple organ systems, including respiratory, cardiovascular, neurological, and gastrointestinal domains. Frequently reported manifestations include fatigue, dyspnea, chest pain, musculoskeletal discomfort, and cognitive impairment. Existing evidence implicates immune dysregulation, sustained inflammatory signaling, metabolic disturbance, and potentially viral persistence or autoimmune processes; however, their relative contributions and interactions remain incompletely resolved (Raveendran et al., 2021).

Epidemiological analyses highlight both the prevalence and heterogeneity of Long COVID. Meta-analytic estimates suggest that approximately 36% of individuals with confirmed SARS-CoV-2 infection experience persistent symptoms, with substantial variation according to disease severity and population characteristics (Hou et al., 2025). Symptoms have been documented in both hospitalized and non-hospitalized individuals, as well as in pediatric cohorts, with persistence extending up to 1 year after infection (CDC, 2026; Koc et al., 2022). This diversity of clinical trajectories underscores the limitations of single-cohort analyses and motivates the need for analytical approaches capable of extracting stable molecular structure across disparate datasets.

In parallel, a wide range of pharmacological and non-pharmacological interventions is under investigation, reflecting ongoing uncertainty regarding the underlying molecular control architecture. Candidates include metabolic and anti-inflammatory agents such as anhydrous enol-oxaloacetate (Cash and Kaufman, 2022), atorvastatin (INSPIRATION-S Investigators, 2022), ivermectin (Fernández-de Las-Peñas et al., 2024), methylnicotinamide (Chudzik et al., 2022), montelukast (Mera-Cordero et al., 2022), and sodium pyruvate nasal formulations (Lupfer et al., 2021). Combination and immunomodulatory approaches, including low-dose naltrexone with NAD supplementation (Isman et al., 2024), corticosteroid–antihistamine nasal sprays (Dietz and Brondstater, 2024), aerosolized gas formulations (Antar and Cox, 2024), and JAK inhibition with baricitinib (Ely and Center, 2025), are also under evaluation. The breadth of these strategies reflects the absence of consensus on dominant molecular mechanisms and highlights the importance of integrative approaches capable of connecting molecular observations to therapeutic hypotheses (Ceban et al., 2022).

Machine learning methods have become increasingly prominent in systems biology as tools for analyzing high-dimensional data and integrating information across biological scales. Prior work has demonstrated their utility for identifying molecular patterns, prioritizing drug targets, and modeling clinical outcomes across diverse biomedical contexts (Zhao et al., 2022; Miao et al., 2020; Vamathevan et al., 2019; Zhang et al., 2025). Related studies show that meaningful structure can be recovered even from limited cohorts, provided that model complexity, overfitting, and cohort-specific effects are carefully controlled (Lin and Lane, 2017; Nishiwaki et al., 2021; Zh et al., 2025).

Nevertheless, many applications in microbiology and immunology prioritize predictive performance within individual datasets, often at the expense of reproducibility and biological consistency across studies. For integrative systems microbiology, the primary objective is not classification accuracy alone, but the identification of stable, interpretable molecular systems that persist across cohorts, tissues, and experimental settings.

Here, we address this challenge by framing molecular inference as a multidataset systems integration problem. We analyze five independent transcriptomic datasets derived from peripheral blood mononuclear cells, whole blood, plasma, and post-mortem tissue, and apply the Matthews Correlation Coefficient Recursive Ensemble Feature Selection (MCC-REFS) algorithm to identify multigene structure that is robust to cohort variability. MCC-REFS is evaluated against alternative feature-selection strategies, and generalization is assessed using independent validation cohorts. The resulting seven-gene system (*PPP2CB*, *SOCS3*, *ARG1*, *FZD2*, *IL6R*, *TRGV3/5*, and *ECHS1*) reflects coordinated immune and metabolic expression patterns. By integrating pharmacological and clinical trial knowledge bases, we contextualize these molecular systems within ongoing therapeutic research. Although Long COVID motivates this analysis, the proposed framework is broadly applicable to integrative systems microbiology problems requiring data-driven interpretation of biological patterns from heterogeneous, multi-source omics data.

## 2 Methods and materials

### 2.1 Algorithmic framework for multi-dataset systems inference

To address the problem of unstable molecular inference across heterogeneous biological datasets, we adopted the Matthews Correlation Coefficient–Recursive Ensemble Feature Selection (MCC-REFS) algorithm as the core analytical framework for this study. MCC-REFS is an ensemble-based feature-selection strategy designed to prioritize reproducibility and cross-dataset generalization rather than single-cohort predictive performance. In this framework, multi-omics integration is achieved through the joint analysis of molecular expression data together with pharmacological and intervention knowledge layers. The method was implemented in Python and is specifically suited for integrative *omics* applications characterized by class imbalance, limited sample sizes, and batch heterogeneity (Rojas-Velazquez et al., 2025a).

MCC-REFS builds upon the Recursive Ensemble Feature Selection (REFS) methodology (Rojas-Velazquez et al., 2024; Peralta-Marzal et al., 2024; Rojas-Velazquez et al., 2025b), which has been previously validated across a range of high-dimensional biological domains, including miRNA (Lopez-Rincon et al., 2019; Lopez-Rincon et al., 2020), mRNA (Kidwai et al., 2023; Metselaar et al., 2021; Rojas-Velazquez et al., 2023), and microbiome profiling (Kamphorst et al., 2023; Benner et al., 2021; Blankestijn et al., 2022; Rojas-Velazquez et al., 2024; Peralta-Marzal et al., 2024; Rojas-Velazquez et al., 2025b). REFS operates by iteratively ranking features through an ensemble of classifiers and recursively eliminating low-ranking variables, thereby emphasizing consensus signals that persist across modeling assumptions. Compared with conventional feature-selection approaches such as Univariate Feature Selection (Lazo and Rathie, 1978), Recursive Feature Elimination (Guyon et al., 2002), Elastic Net (Sokolov et al., 2016), Genetic Algorithms (Trevino and Falciani, 2006), LASSO (Tibshirani, 1996), and Ensemble Feature Selection with Complete Linear Aggregation (Abeel et al., 2010), REFS has shown improved robustness in noisy, high-dimensional biological settings.

MCC-REFS extends REFS by incorporating Matthews Correlation Coefficient (MCC) as the optimization criterion, ensuring balanced performance under class imbalance while promoting stability of selected feature subsets. The method integrates eight classifiers from the *scikit-learn* library (Pedregosa et al., 2011):

- Gradient Boosting (n_estimators=300)
- Random Forest (n_estimators=300)
- Logistic Regression
- Passive Aggressive
- Stochastic Gradient Descent (SGD)
- Support Vector Machine with linear kernel
- Ridge
- Bagging (n_estimators=300)

All remaining hyperparameters were kept at their default values from the *scikit-learn* library, following the principle of combining diverse, minimally biased algorithms in the ensemble. Each contributing a ranked feature list at every iteration. These rankings are aggregated to enforce ensemble consensus. A key property of MCC-REFS is its ability to automatically determine the optimal number of retained features (k) , avoiding arbitrary thresholding and enabling self-regularization across datasets. In contrast, alternative approaches such as GRACES (Chen et al., 2023), SHAP (Lundberg and Lee, 2017) and Deep Neural Pursuit (DNP) (Liu et al., 2017) require manual or heuristic specification of feature-set size.

### 2.2 Nested validation and stability analysis

To ensure strict separation between model selection and performance estimation, all analyses were conducted within a fully nested cross-validation framework (Vabalas et al., 2019). This design prevents information leakage and is particularly critical in small-sample, high-dimensional settings common to multi-cohort *omics* studies. We evaluated the algorithm over 10 runs of 10-fold stratified cross-validation, implemented with *scikit-learn’s StratifiedKFold* to compensate for class imbalance across folds^1^.

Reproducibility of the inferred molecular structure was evaluated through stability analysis across 50 resampled runs. Pairwise Jaccard similarity coefficients and the Nogueira stability index were computed to quantify the consistency of selected feature sets (Nogueira and Brown, 2016), providing a systems-level assessment of whether the identified gene subsets reflect robust biological signals rather than sampling variability. To complement this stability-based evaluation, MANOVA was applied after feature reduction to assess the statistical significance of multivariate group separation induced by the selected feature set (Pérez-Cova et al., 2022). Multiple testing corrections were applied exclusively to univariate follow-up analyses, with the Benjamini–Hochberg procedure used to control the false discovery rate in an exploratory setting suitable for high-dimensional feature screening (Arici et al., 2025), and Bonferroni correction reported as a more conservative approach to ensure rigorous control of Type I error (VanderWeele and Mathur, 2019). While univariate follow-up analyses are reported, the focus of this study is on multivariate system-level structure rather than individual effect sizes, which may be unstable in high-dimensional settings due to feature interdependencies (Rahnenführer et al., 2023).

For methodological benchmarking, MCC-REFS was evaluated alongside GRACES (Chen et al., 2023), Recursive Feature Elimination (Guyon et al., 2002), SelectKBest (Scikit-learn Developers, 2026a), and DNP (Liu et al., 2017), Random Forest Feature Importance (Scikit-learn Developers, 2026b), Elastic Net (Sokolov et al., 2016), Boruta (Kursa et al., 2010), LASSO (Tibshirani, 1996) and SHAP (Lundberg and Lee, 2017).

GRACES employs repeated single-feature selection across multiple random seeds followed by frequency-based aggregation to obtain a stable subset of size k (Chen et al., 2023). At each iteration, a single feature is selected using a graph-based neural model with key hyperparameters including the number of dropout passes, dropout probability, learning rate, number of epochs, and graph sparsity control, while gradient norms guide selection and an optional F-test correction stabilizes ranking. The procedure is repeated iteratively until k features are obtained per run, and final selection is performed by ranking features according to their frequency across seeds. DNP constructs compact feature subsets via greedy forward selection, iteratively activating features based on input-layer gradient group norms averaged across multiple dropout realizations, while constraining all unselected input weights to zero during training to prevent information leakage (Liu et al., 2017). SHAP-based selection computes feature relevance by averaging absolute Shapley values derived from a tree-based ensemble and subsequently retains the top- k features according to their importance (Lundberg and Lee, 2017). Boruta performs all-relevant feature selection by iteratively comparing real features against permuted shadow variables within a random forest framework, retaining only those features whose importance is consistently higher than shadow features, thereby defining an implicit cutoff without requiring predefined k (Kursa et al., 2010).

Elastic Net selection identifies informative variables through regularized logistic regression with combined $\ell_{1}$ and $\ell_{2}$ penalties, selecting features with non-zero coefficients after optimization (Sokolov et al., 2016). LASSO constitutes a purely sparse variant using only $\ell_{1}$ regularization, shrinking irrelevant coefficients exactly to zero and implicitly determining the selected subset (Tibshirani, 1996). Recursive Feature Elimination with cross-validation (RFECV) performs iterative backward elimination and automatically determines the optimal number of features based on cross-validated performance (Guyon et al., 2002). SelectKBest performs univariate feature ranking using statistical criteria and explicitly retains the top- k features, requiring the subset size as a hyperparameter (Scikit-learn Developers, 2026a). Random Forest importance-based selection ranks variables according to impurity-based importance scores derived from an ensemble of decision trees and similarly retains the top- k features, requiring predefined specification of k (Scikit-learn Developers, 2026b). Together, these methods provide complementary baselines spanning a wide range of feature selection strategies. Formal statistical comparisons of MCC-REFS with alternative feature-selection methods have been previously established in benchmarking studies (Rojas-Velazquez et al., 2024; Rojas-Velazquez et al., 2025a; Rojas-Velazquez et al., 2025b; Lopez-Rincon et al., 2019).

Given the multi-dataset nature of this study, batch effects and platform variability were addressed through z-score normalization and by emphasizing methods that prioritize relative feature importance over absolute expression levels. MCC-REFS inherently mitigates batch-specific artifacts through ensemble aggregation and cross-validation. Furthermore, validation across datasets derived from different tissues and sequencing technologies (RNA-seq, NanoString, post-mortem expression profiling) serves as an implicit test of robustness to batch heterogeneity. We therefore treat cross-context generalization as an explicit systems-level evaluation criterion rather than a limitation.

For downstream performance evaluation, we deliberately employed a classifier that was not included in the MCC-REFS ensemble to avoid implicit overfitting to the selection process (Ying, 2019). Specifically, a Multi-layer Perceptron (MLP) classifier (Bishop, 1995) with default parameters was trained using 10-fold cross-validation. Default hyperparameters were retained to demonstrate that the inferred feature systems exhibit discriminative structure without model-specific tuning. The MLP classifier was used with the default configuration of the scikit-learn implementation, consisting of a single hidden layer with 100 neurons, ReLU activation, the Adam optimizer, a maximum of 200 iterations, and default settings for learning rate, regularization, and batch size.

### 2.3 Integration of pharmacological and interventional knowledge bases

To support mechanistic interpretation of the inferred gene systems, we implemented a reproducible knowledge-integration pipeline that maps selected genes to pharmacological and interventional evidence. Using a custom Python workflow, each ENSG identifier was queried against *Open Targets* (Koscielny et al., 2017), *DGIdb* (Cannon et al., 2024), and *DrugBank* (Knox et al., 2024) to retrieve known drug associations, mechanisms of action, approval status, and pharmacogenomic evidence. Data from these resources were standardized, deduplicated, and merged into unified gene-centric summary tables, enabling systematic comparison across databases.

**FIGURE 1:**
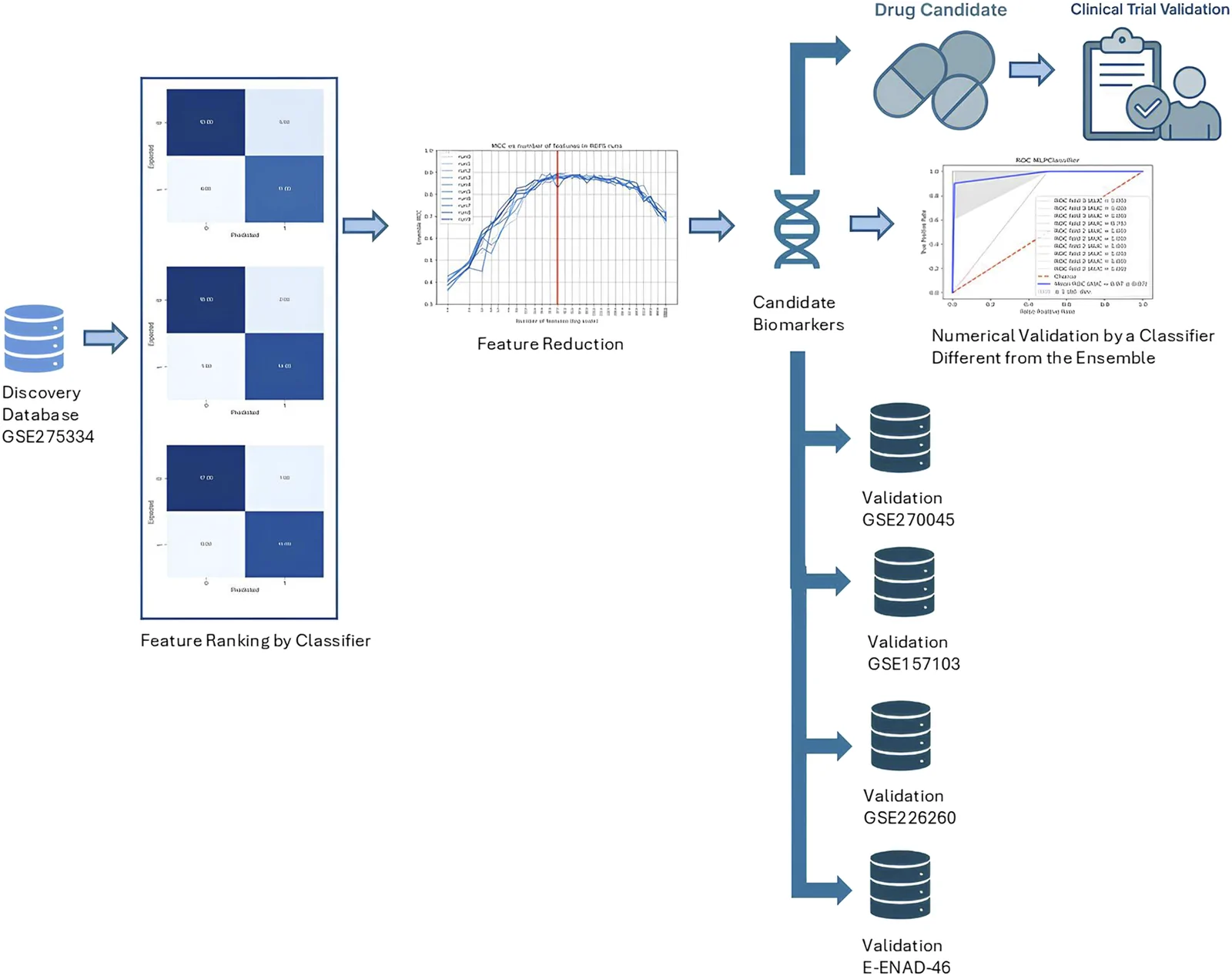
Algorithmic framework for multi-dataset systems inference and external validation. MCC-REFS is applied to the discovery dataset and evaluated across independent cohorts and knowledge layers. The genes are found in GSE275334 discovery dataset with MCC-REFS and validated in clinical trials and in GSE270045, GSE226260, GSE157103 and E-ENAD-46 validation datasets.

To extend this integration to the interventional layer, we automatically queried the ClinicalTrials.gov (Zarin et al., 2011) API for each identified drug across the conditions “Long COVID,” “COVID-19,” “COVID,” and “SARS-CoV-2.” Each retrieved trial entry was programmatically verified to ensure relevance to the queried compound. The resulting structured tables provide a reproducible overview of existing clinical activity linked to the inferred gene systems. This integration step is not intended as clinical validation, but rather as a systems-level mapping that connects molecular inference to ongoing translational efforts. The full algorithmic workflow is provided in Annex 1 (Supplementary Algorithm S1). A visual explanation of the pipeline is in Figure 1.

### 2.4 GSE275334 (discovery dataset)

The dataset GSE275334 (Eaton-Fitch et al., 2024), “*Immune Exhaustion in ME/CFS and long COVID*” involves gene expression analysis using the NanoString nCounter Immune Exhaustion gene expression panel. This panel includes 785 genes to investigate mechanisms behind T cell, B cell, and NK cell exhaustion in disease. RNA was extracted from peripheral blood mononuclear cells (PBMCs) from participants with ME/CFS (n = 14), long COVID (n = 15), and healthy controls (n = 18). The study aimed to compare gene expression profiles between these groups to understand immune exhaustion in ME/CFS and long COVID. The data was normalized against controls to account for background noise and platform variation, and differential expression was reported between ME/CFS, long COVID, and healthy controls as reported by the author. As discovery set, we only used long COVID patients (n = 15) and healthy controls (n = 18). We used the data as provided in the file *“GSE275334_File_1_Normalised.xlsx”*, available at the Gene Expression Omnibus (GEO) repository^2^. This dataset was selected as the discovery cohort because it has the smallest feature space among the available datasets, following the procedure described in (Rojas-Velazquez et al., 2024), where feature selection is first performed on the lowest-dimensional dataset to improve stability.

### 2.5 GSE270045 (validation dataset)

The dataset GSE270045 (Rahmati et al., 2025), “*Upregulation of olfactory receptors and neuronal-associated genes highlights complex immune and neuronal dysregulation in Long COVID patients*” involves gene expression analysis using high throughput sequencing. RNA was isolated from the whole blood of 19 long COVID patients who developed myalgic encephalomyelitis/chronic fatigue syndrome (ME/CFS) following acute SARS-CoV-2 infection, and from 17 healthy controls (HCs). The study aimed to investigate the differential gene expression patterns between these groups to understand the immune and neuronal dysregulation in long COVID patients. We used the data as provided in the file *“GSE270045_LC_counts.tsv”*, available at the Gene Expression Omnibus (GEO) repository^3^.

### 2.6 GSE226260 (validation dataset)

The dataset GSE226260 (Aid and Barouch, 2023; Aid et al., 2025), *“Persistent Activation of Chronic Inflammatory Pathways in Long Covid”*, is a peripheral blood RNA-sequencing study designed to characterize chronic immune dysregulation associated with post-acute sequelae of SARS-CoV-2 infection (PASC). In the present analysis, we exclusively used samples contained in the *raw counts* file, which includes expression profiles from 331 samples. We created a subset based on the available labels. This subset consists of 124 Long COVID (PASC) cases and 63 controls (Recovered/No PASC). RNA was derived from PBMCs, and the study aimed to identify persistent inflammatory and immune-related transcriptional signatures distinguishing Long COVID patients from recovered individuals. This subset therefore serves as an independent validation cohort focused on chronic immune activation following SARS-CoV-2 infection. We used the data as provided in the files, available at the Gene Expression Omnibus (GEO) repository^4^.

- *“GSE226260_AdditionalSamples.rawCounts.csv”*
- *“GSE226260_combined.ExprData.updated”*

### 2.7 GSE157103 (external validation dataset)

The dataset GSE157103 (Overmyer et al., 2021), “*Largescale Multi-omic Analysis of COVID-19 Severity*” involves a comprehensive systems analysis using RNA-seq and high-resolution mass spectrometry. The study analyzed 128 plasma and leukocyte samples from hospitalized patients with or without COVID-19 (n = 102 and 26, respectively) and with varying degrees of disease severity. The aim was to generate abundance measurements for over 17,000 transcripts, proteins, metabolites, and lipids, and compile them with clinical data into a curated relational database. As validation set, we compared ICU (n = 50) against non-ICU COVID patients (n = 50). We used the data as provided in the file *“GSE157103_genes.ec.tsv”* and we only kept the ones with the label *COVID*, *ICU* or *NonICU*, available at the Gene Expression Omnibus (GEO) repository^5^.

### 2.8 E-ENAD-46 (external validation dataset)

To assess whether the identified Long COVID gene signature captures biological processes extending beyond circulating immune compartments, we evaluated its performance in an independent tissue-level RNA-sequencing dataset of fatal COVID-19 cases (E-ENAD-46). This dataset includes post-mortem lung and colon samples from deceased COVID-19 patients and non- COVID controls.

The E-ENAD-46 dataset, curated within the EMBL-EBI Expression Atlas, provides RNA-sequencing–based transcriptomic profiles of post-mortem lung and colon tissues from fatal COVID-19 cases (Wu et al., 2020). COVID-19 samples are contrasted against non- COVID-19 post-mortem controls, enabling the assessment of tissue-specific host transcriptional responses associated with severe SARS-CoV-2 infection. Gene-level expression data are released as raw sequencing counts, supporting differential expression and biomarker discovery analyses. We used the data as provided in the file *“E-ENAD-46-raw-counts.tsv”* and we matched the IDs in the file *“E-ENAD-46-experiment-design.tsv”*, available at the EMBL-EBI repository, using the labels *COVID* or *normal* ^6^. Given that E-ENAD-46 was generated early in the pandemic (2021) and includes fatal COVID-19 cases, it can be interpreted as a proxy for severe disease.

A summary of all the available datasets is in Table 1. Each dataset was preprocessed following a consistent pipeline. First, missing values were handled when necessary using mean imputation, where absent entries were replaced by the average value of the corresponding feature. Subsequently, feature scaling was applied using z-score standardization. To ensure robustness and avoid information leakage, the z-score normalization was performed within a 10-fold cross-validation framework, computing the mean and standard deviation exclusively on the training folds and applying the transformation to the corresponding validation fold within each database.

**TABLE 1:** Transcriptomic datasets used in this study, including sample type and case/control comparisons.

Dataset ID	Role in study	Sample type	Comparison (cases/controls)
GSE275334	Discovery	PBMCs	Long COVID vs. healthy controls (15/18)
GSE270045	Validation	Whole blood	Long COVID vs. healthy controls (19/17)
GSE226260	Validation	PBMCs	Long COVID (PASC) vs. recovered (63/124)
GSE157103	External validation	Plasma and leukocytes	ICU vs. non-ICU COVID-19 patients (50/50)
E-ENAD-46	External validation	Post-mortem tissue (lung and colon)	Fatal COVID-19 vs. non-COVID controls (18/20)

Some datasets reflect acute COVID, enabling comparison across multiple stages of SARS-CoV-2 disease rather than Long COVID alone.

## 3 Results

### 3.1 GSE275334 (discovery dataset)

**FIGURE 2:**
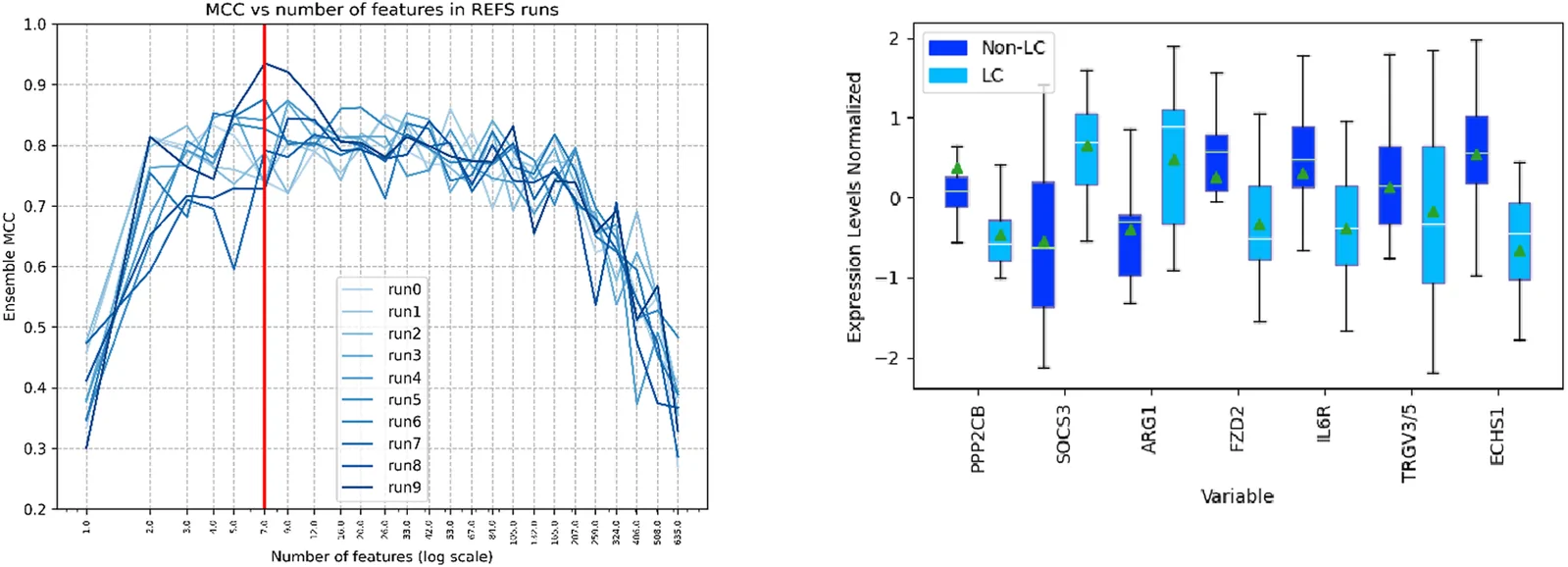
(Left) Gene reduction from 635 to 7 using MCC-REFS in GSE275334. (Right) Boxplot comparison of expression levels for selected genes.

Using the raw counts from dataset GSE275334 and samples for long COVID (n = 15), and healthy controls (n = 18), we applied MCC-REFS and reduced it from 635 to 7 genes (*PPP2CB*, *SOCS3*, *ARG1*, *IL6R*, *ECHS1*, *FZD2*, *TRGV3/5*). TRGV3/5 denotes a TRGV3/TRGV5 family probe in the discovery dataset. Because the platform does not distinguish TRGV3 from TRGV5, the feature is reported as TRGV3/5. When available in validation datasets, TRGV3 and TRGV5 were analyzed separately. We calculate the ROC curve using the MLP classifier, a classifier not part of the ensemble, to avoid over-fitting (Figure 2).

Multivariate Analysis of Variance (MANOVA) (IBM Corporation, 2011) was conducted on the seven-gene expression panel to compare individuals with Long COVID (n = 15) and healthy controls (n = 18). The analysis revealed a highly significant multivariate effect of disease status, indicating that the combined gene expression profile robustly differentiates the two groups. This result was consistently supported across all multivariate test statistics, with Wilks’ Lambda = 0.1594, Pillai’s Trace = 0.8406, Hotelling–Lawley Trace = 5.2722, and Roy’s Greatest Root = 5.2722. The overall multivariate test was highly significant (F (7,26) = 19.58, p < 0.0001), demonstrating that the observed group differences in gene expression are extremely unlikely to be due to chance.

Follow-up univariate analyses clarified the individual gene contributions driving this multivariate effect. *SOCS3* and *ARG1* were significantly overexpressed in Long COVID, while *PPP2CB* and *ECHS1* were significantly underexpressed relative to healthy controls (p < 0.05). *IL6R* showed a modest decrease in expression in Long COVID but did not contribute as strongly, whereas *FZD2* and *TRGV3/5* showed no significant differential expression. Together, these results indicate that the strong multivariate separation observed is primarily driven by dysregulation of *SOCS3*, *ARG1*, *PPP2CB*, and *ECHS1*, with the remaining genes contributing weakly or not significantly at the univariate level.

**FIGURE 3:**
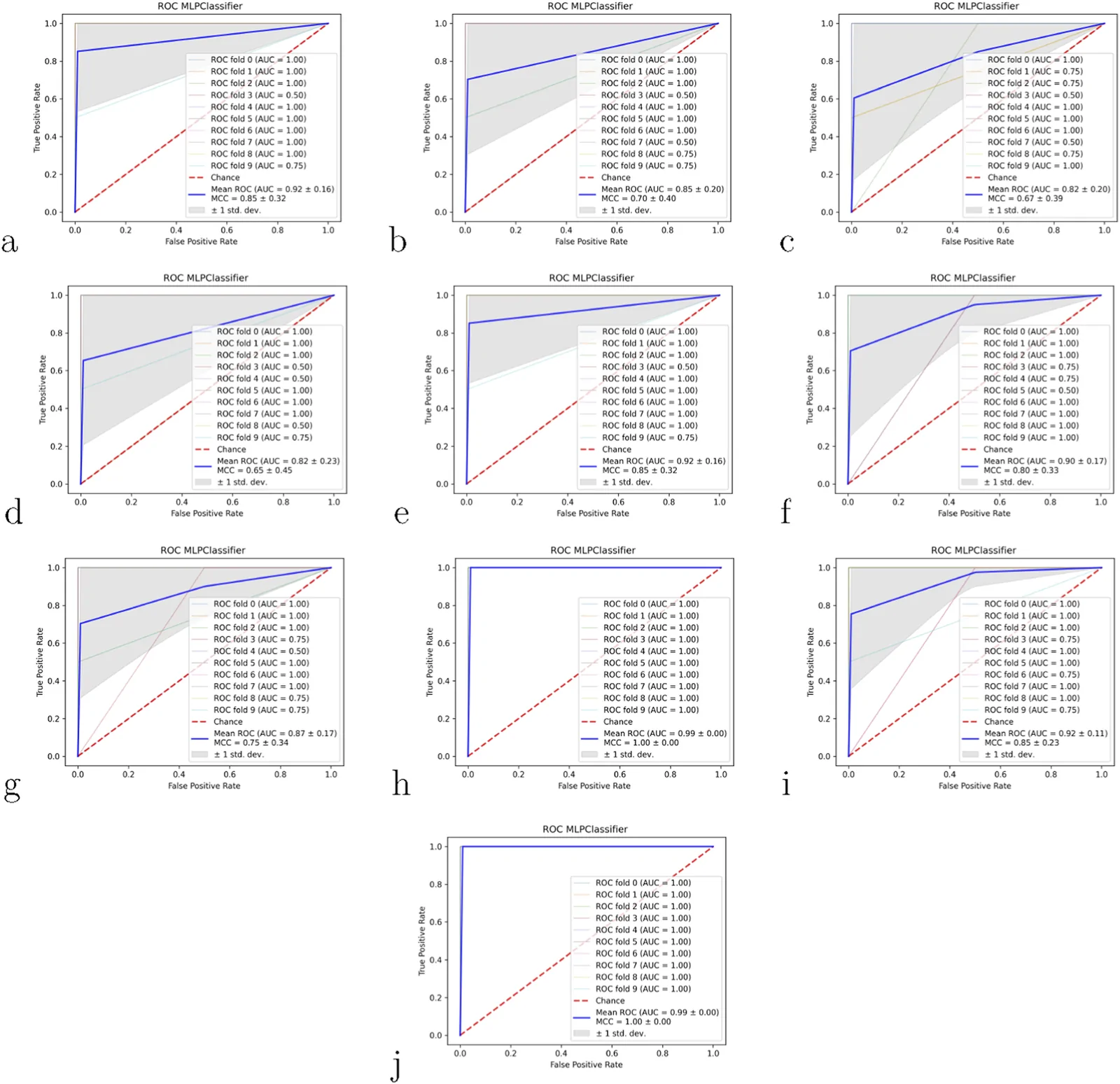
AUC obtained from 10-fold cross-validation of MLP classifiers with default parameters using features selected by different methods in GSE275334. Figure **(a)** presents the results for Elastic Net (EN) 92 selected features, **(b)** Boruta 17 features, **(c)** DNP 7 features, **(d)** GRACES 7 features, **(e)** SelectKBest (F-score) 7 features, **(f)** LASSO 12 features, **(g)** Random Forest feature importance 7 features, **(h)** RFECV 67 features, **(i)** SHAP-based feature selection 7 features, and **(j)** MCC-REFS 7 features. Random seeds were fixed for comparison and reproducibility.

Next, we compared MCC-REFS with several established feature-selection methods, including SelectKBest (F-score, k=7 ), DNP (k=7) , GRACES (k=7) , Random Forest feature importance (RF, k=7 ), SHAP (k=7) , and RFE-CV, Elastic Net (EN), LASSO, and BORUTA, which automatically determine the number of selected features (Figure 3). The genes selected by each method are summarized in Table 2. Using the selected features as input to an MLP classifier with default parameters, MCC-REFS achieved the best overall performance, reaching an AUC of 0.99±0.00 and an MCC of 1.00±0.00 . Although RFE-CV obtained the same predictive performance, it required a substantially larger feature set (67 genes versus 7 genes for MCC-REFS). Similarly, Elastic Net selected 92 genes but achieved lower performance (AUC = 0.92±0.16 , MCC = 0.85±0.32 ). SHAP, SelectKBest, LASSO, RF, BORUTA, DNP, and GRACES all yielded lower AUC and MCC values despite selecting a similar or greater number of features. Notably, DNP and GRACES, which were specifically designed for high-dimensional, low-sample-size datasets, achieved AUC values of only 0.82±0.20 and 0.82±0.23 , respectively. Overall, these results demonstrate that MCC-REFS provides a compact seven-gene signature that achieves near-perfect classification performance while maintaining a substantially smaller feature set than most competing approaches.

**TABLE 2:** Comparison of feature-selection methods on the GSE275334 long COVID versus healthy control discovery cohort.

Method	PPP2CB	SOCS3	ARG1	IL6R	ECHS1	FZD2	TRGV3/5	# Features	AUC	MCC
MCC-REFS	✓	✓	✓	✓	✓	✓	✓	7	0.99±0.00	1.00±0.00
RFE-CV	✓	✓	✓	✓	✓	✓	✓	67	0.99±0.00	1.00±0.00
SHAP	✓	–	–	✓	✓	–	–	7	0.92±0.11	0.85±0.23
KBEST	–	✓	–	–	✓	–	–	7	0.92±0.16	0.85±0.32
EN	✓	✓	✓	✓	✓	✓	✓	92	0.92±0.16	0.85±0.32
LASSO	–	✓	✓	–	✓	✓	–	12	0.90±0.17	0.80±0.33
RF	✓	✓	–	–	✓	–	–	7	0.87±0.17	0.75±0.34
BORUTA	✓	✓	–	–	✓	–	–	16	0.85±0.20	0.70±0.40
DNP	–	✓	✓	–	–	–	–	7	0.82±0.20	0.67±0.39
GRACES	–	✓	–	–	✓	–	–	7	0.82±0.23	0.65±0.45

The table reports the presence of MCC-REFS genes, the number of selected features, and MLP classification performance (AUC and MCC, mean ± SD). MLP classifier with default parameters.

The feature-selection process shows moderate-to-strong overall stability, with a mean pairwise Jaccard index of 0.613 and a Nogueira stability of 0.558, reflecting consistent overlap in the selected subsets across 50 cross-validation runs. On average, the selector retained 4.18 ± 0.71 features out of 7, and the downstream classifier, indicating strong predictive performance even as feature subsets vary. At the feature level, *SOCS3* and *ARG1* were selected in 100% of runs, making them the most stable markers and also selected by DNP, while *PPP2CB* showed high but slightly lower stability at 80%. The remaining features, *IL6R*, *TRGV3/5*, *ECHS1*, and *FZD2*, were selected less consistently ( ≤ 42%), suggesting they may be context-dependent or weaker contributors. Together, these results indicate that the dataset contains a core set of highly stable biomarkers, supported by a moderately consistent feature-selection pattern, which is commendable given the small sample size and the inherent variability of cross-validated L1-regularized methods. Although the observed stability metrics are moderate, they are consistent with expectations for high-dimensional, small-sample omics data. A more detailed analysis of stability behavior and comparisons with alternative feature-selection methods is provided in prior methodological work on MCC-REFS. These results confirm that despite the small sample size, the stability of feature selection (Jaccard = 0.613; Nogueira = 0.558) and strict methodological separation mitigate the risk of overfitting and support the reproducibility of the MCC-REFS signature.

### 3.2 GSE270045 (validation dataset)

Multivariate Analysis of Variance (MANOVA) applied to the seven-gene expression panel revealed a statistically significant multivariate effect of disease status, confirming that the combined gene expression profile differentiates Long COVID patients with ME/CFS from healthy controls. This effect was consistently supported across all multivariate test statistics (Wilks’ Lambda = 0.4205, Pillai’s Trace = 0.5795, Hotelling–Lawley Trace = 1.3780, and Roy’s Greatest Root = 1.3780). The overall model was highly significant (F (7,29) = 5.71, p = 0.0003), indicating that a substantial proportion of variance in the multigene expression space is attributable to disease status rather than random variation.

**FIGURE 4:**
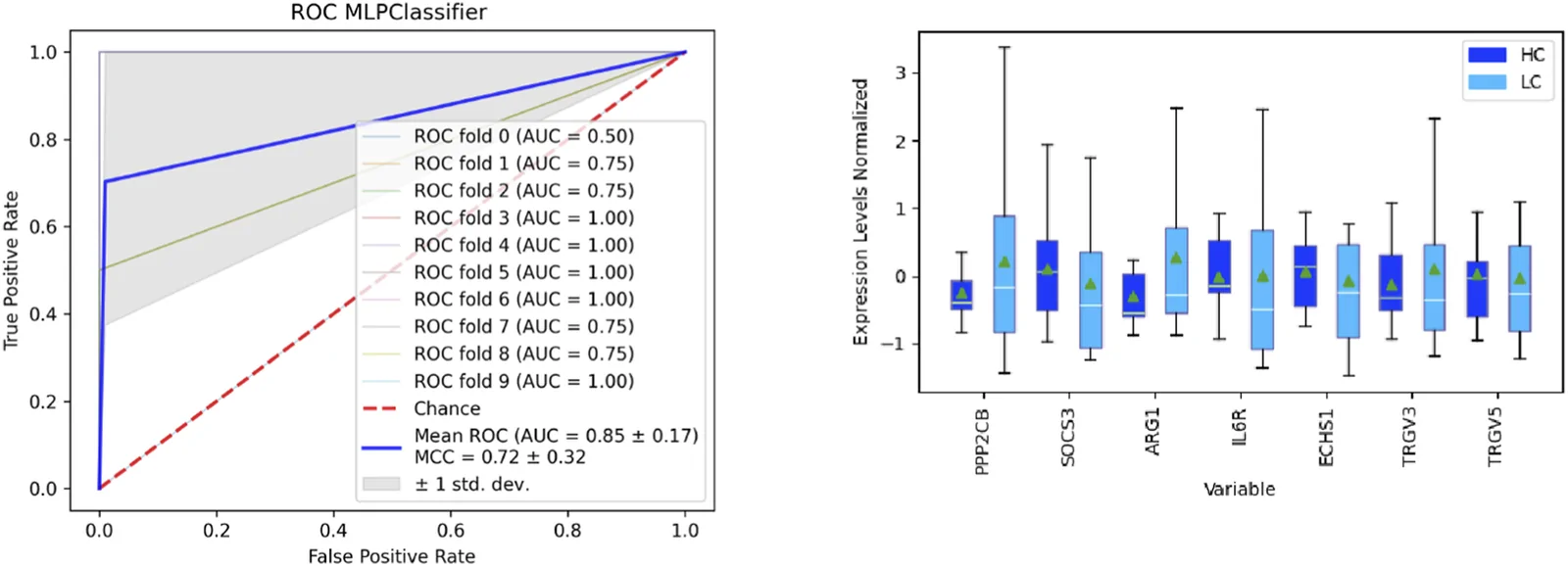
(Left) AUC using MLP 10-fold cross-validation of dataset GSE270045 with the available genes, 6 of 7. (Right) Box-plot of the genes.

To assess predictive performance, we applied a Multi-Layer Perceptron (MLP) classifier under 10-fold cross-validation, achieving a mean area under the ROC curve (AUC) of 0.85±0.17 , which reflects strong diagnostic accuracy for this gene panel (Figure 4).

Follow-up univariate analyses showed that no individual gene remained statistically significant after correction for multiple testing, suggesting that the observed group separation is driven by a coordinated multigene expression pattern rather than a single dominant marker. Directionally, *ARG1*, *PPP2CB*, *IL6R*, and *TRGV3* exhibited higher expression in Long COVID/ME-CFS, whereas *SOCS3*, *ECHS1*, and *TRGV5* showed lower expression compared with healthy controls. Although *ARG1* and *PPP2CB* demonstrated the strongest univariate trends, these effects were modest, underscoring the importance of the multivariate gene expression signature in characterizing Long COVID–associated ME/CFS.

### 3.3 GSE226260 (validation dataset)

Multivariate Analysis of Variance (MANOVA) applied to the eight-gene expression panel revealed a significant multivariate effect of disease status, confirming that the combined expression profile discriminates Long COVID (PASC) patients from recovered and no PASC controls. This effect was consistently supported across all multivariate test statistics (Wilks’ Lambda = 0.5518, Pillai’s Trace = 0.4482, Hotelling–Lawley Trace = 0.8123, and Roy’s Greatest Root = 0.8123), with the overall model highly significant (F (8,95) = 9.65, p < 1.07e-09), indicating that group differences are unlikely to be due to chance.

**FIGURE 5:**
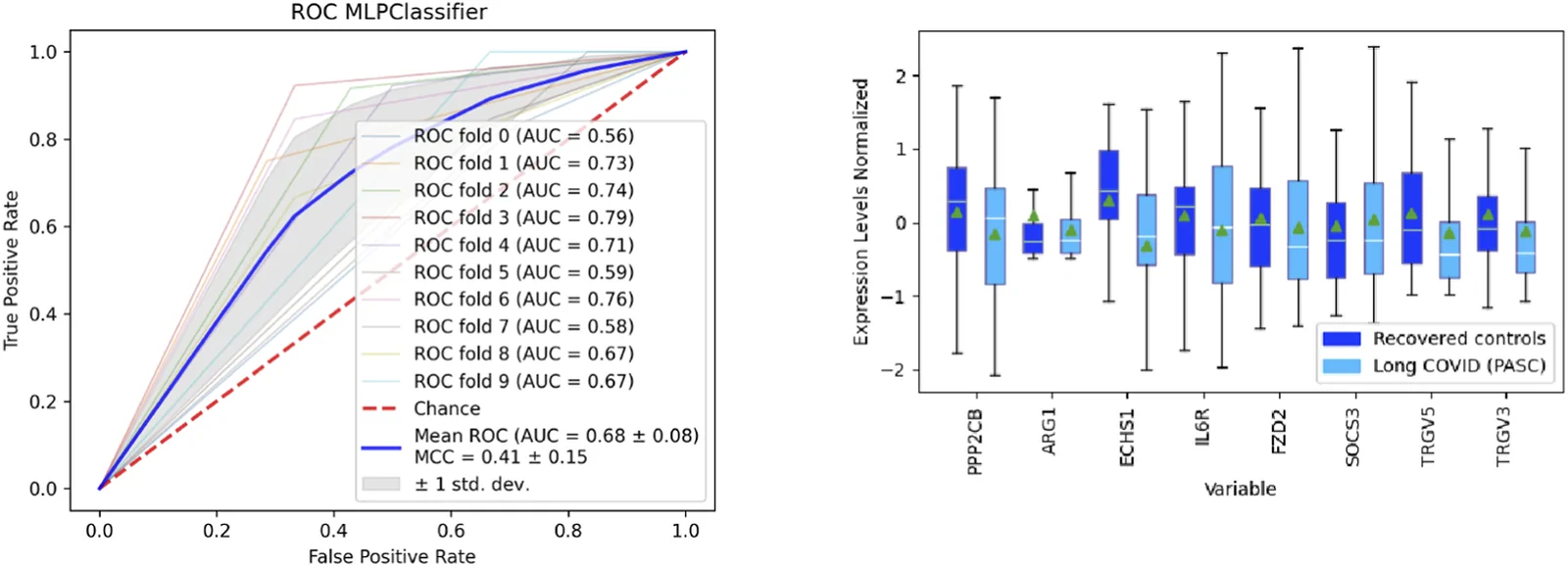
(Left) AUC using MLP 10-fold cross-validation of dataset GSE226260. (Right) Box-plot of the genes.

To evaluate predictive performance, we applied a Multi-Layer Perceptron (MLP) classifier under 10-fold cross-validation, achieving a mean area under the ROC curve (AUC) of 0.68±0.08 (Figure 5). Although lower than in other cohorts, this value reflects moderate diagnostic accuracy for distinguishing Long COVID from recovered individuals based on the multigene signature.

Univariate follow-up analyses identified *ECHS1* as the only gene remaining significant after correction for multiple testing (p = 0.0015), with lower expression observed in Long COVID cases compared with controls. The remaining genes showed only modest, non-significant differences, reinforcing that disease-associated separation is primarily driven by a coordinated multigene expression pattern rather than strong effects of individual genes.

### 3.4 GSE157103 (external validation dataset)

Multivariate Analysis of Variance (MANOVA) applied to the six-gene expression panel revealed a highly significant effect of ICU status, confirming that the combined expression profile robustly discriminates ICU COVID-19 patients from non-ICU patients. This effect was consistently supported across all multivariate test statistics (Wilks’ Lambda = 0.3182, Pillai’s Trace = 0.6818, Hotelling–Lawley Trace = 2.1428, and Roy’s Greatest Root = 2.1428), with the overall model extremely significant (F (6,94) = 33.57, p < 2.36e-21), indicating that group differences are unlikely to occur by chance.

**FIGURE 6:**
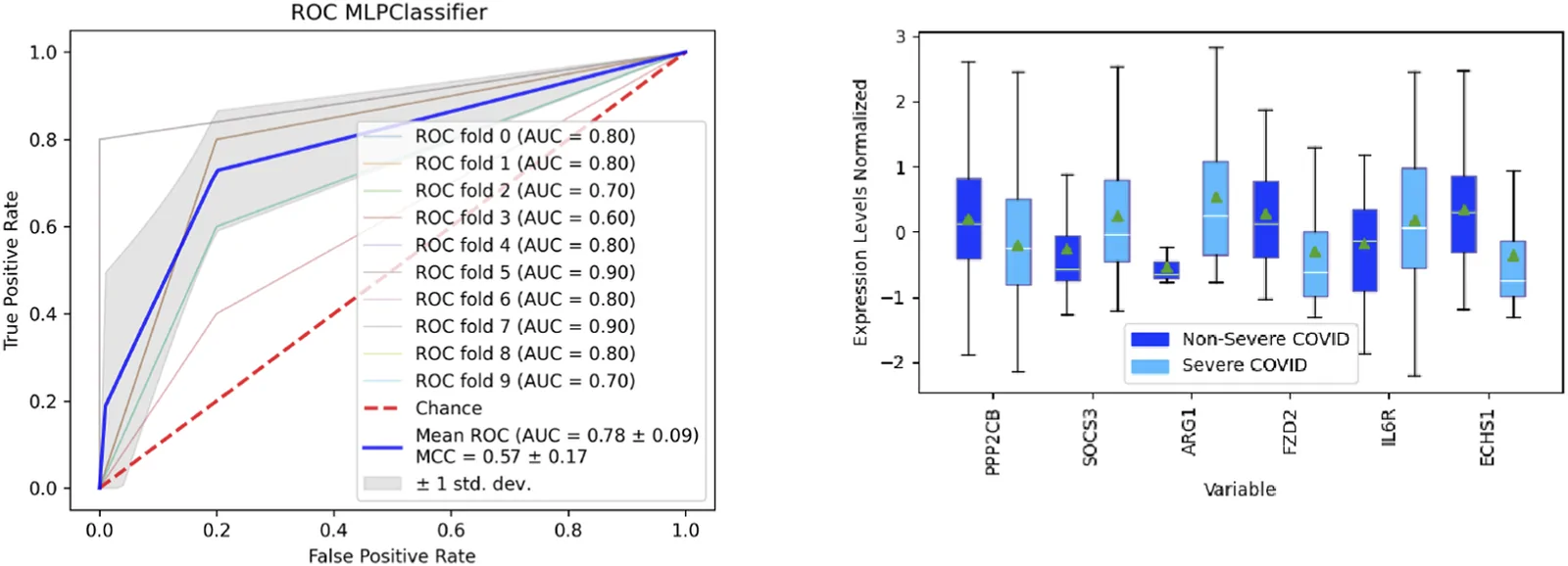
(Left) AUC using MLP 10-fold cross-validation of dataset GSE157103 with the available genes, 6 of 7. (Right) Box-plot of the genes.

To assess predictive performance, we applied a Multi-Layer Perceptron (MLP) classifier under 10-fold cross-validation, achieving a mean area under the ROC curve (AUC) of 0.78±0.09 (Figure 6). This reflects strong diagnostic accuracy for distinguishing ICU from non-ICU COVID-19 patients based on the multigene signature.

Univariate follow-up analyses with correction for multiple testing identified *ARG1*, *ECHS1*, *FZD2*, and *SOCS3* as remaining significant after false-discovery-rate adjustment. *ARG1* showed markedly higher expression in ICU patients, whereas *ECHS1* and *FZD2* were significantly reduced in ICU cases. *SOCS3* was moderately elevated in ICU patients. In contrast, *PPP2CB* and *IL6R* did not remain significant after correction. These results indicate that ICU-associated separation is driven by a coordinated multigene expression pattern, with particularly strong contributions from *ARG1* and consistent directional shifts across several genes rather than uniform effects across the entire panel.

### 3.5 E-ENAD-46 (external validation dataset)

Multivariate Analysis of Variance (MANOVA) applied to the eight-gene expression panel revealed a statistically significant effect of *COVID-19 status*, indicating that the combined transcriptional profile discriminates COVID-19 post-mortem samples from non-COVID controls. This multivariate effect was consistently supported across all omnibus test statistics (Wilks’ Lambda = 0.5524, Pillai’s Trace = 0.4476, Hotelling–Lawley Trace = 0.8103, and Roy’s Greatest Root = 0.8103), with the overall model reaching significance (F (8,30) = 3.04, p=0.0126 ). These findings support coordinated gene-expression alterations associated with severe SARS-CoV-2 infection.

**FIGURE 7:**
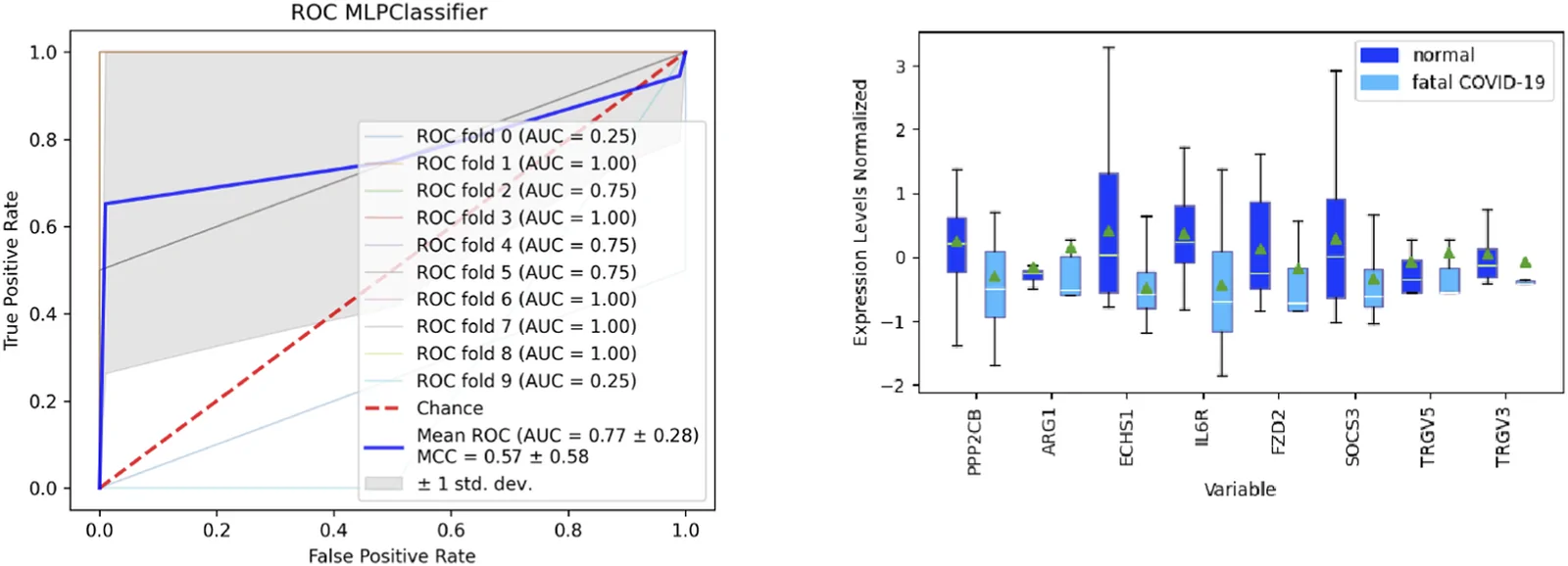
External tissue-level validation (E-ENAD-46) of the Long COVID gene signature. Left: AUC from stratified 10-fold CV MLP using available genes; Right: per-gene boxplots. Reduced performance relative to blood-based datasets reflects tissue compartment and disease-stage differences rather than reduced model robustness.

To evaluate predictive performance, we applied a Multi-Layer Perceptron (MLP) classifier under 10-fold cross-validation, achieving a mean area under the ROC curve (AUC) of 0.77±0.28 (Figure 7). Although this performance is lower than that observed in blood-based datasets, it remains biologically meaningful given the substantial differences in tissue composition, cellular heterogeneity, and disease stage.

Univariate follow-up analyses with correction for multiple testing identified *ECHS1* and *IL6R* as remaining significant after false-discovery-rate adjustment. Both genes exhibited reduced expression in COVID-19 samples compared with controls, suggesting disruption of mitochondrial metabolic function and altered cytokine signaling, respectively. *SOCS3* showed a nominal trend toward differential expression, while *PPP2CB*, *ARG1*, *FZD2*, *TRGV5*, and *TRGV3* were not statistically significant at the corrected level. Together, these results indicate that COVID-associated separation is driven by a coordinated multigene expression pattern rather than uniform effects across the entire panel, with the strongest individual contributions arising from *ECHS1* and *IL6R*.

### 3.6 Cross-dataset consistency of the inferred gene system

The multigene signature demonstrated consistent diagnostic performance across all datasets. Based on mean AUC values of 0.85 (GSE270045), 0.68 (GSE226260), 0.78 (GSE157103), and 0.77 (E-ENAD-46), classification accuracy is considered *very good* for GSE270045, *good* for GSE157103, and E-ENAD-46, and *sufficient* for GSE226260 according to established diagnostic benchmarks (Šimundić, 2009). These findings confirm the robustness of the identified biomarkers beyond the discovery cohort. Because each dataset uses different platforms and gene coverage, not all seven markers are present in every cohort.

Statistical significance for differential expression was assessed using the Benjamini–Hochberg false discovery rate (FDR) (Benjamini and Hochberg, 1995), with Bonferroni-adjusted p-values reported for highly conservative inference (Dunn, 1961). Consistent with multivariate modeling, individual gene-level significance was often modest after correction, indicating that the discriminatory power arises from the coordinated multigene expression pattern rather than from single dominant markers. Table 3 summarizes gene-level expression trends, significance across datasets, MANOVA F-scores, and mean AUC values obtained under 10-fold cross-validation with the MLP classifier.

**TABLE 3:** Expression trends (+ overexpressed, – underexpressed) and significance (n.s., FDR, Bonferroni) across datasets. MANOVA F-scores, AUC and MCC values are also reported.

Gene	GSE275334	GSE270045	GSE226260	GSE157103	E-ENAD-46
PPP2CB	- (FDR)	+ (n.s.)	- (n.s.)	- (n.s.)	- (n.s.)
SOCS3	+ (FDR; Bon.)	- (n.s.)	+ (n.s.)	+ (FDR)	- (n.s.)
ARG1	+ (FDR)	+ (n.s.)	- (n.s.)	+ (FDR; Bon.)	- (n.s.)
FZD2	- (n.s.)	NA	- (n.s.)	- (FDR; Bon.)	- (n.s.)
IL6R	- (n.s.)	+ (n.s.)	- (n.s.)	+ (n.s.)	- (FDR)
ECHS1	- (FDR; Bon.)	- (n.s.)	- (FDR; Bon.)	- (FDR; Bon.)	- (FDR; Bon.)
TRGV3	- (n.s.)	+ (n.s.)	- (n.s.)	NA	- (n.s.)
TRGV5	- (n.s.)	- (n.s.)	- (n.s.)	NA	- (n.s.)
MANOVA	7.23e-09	0.0003	1.07e-09	2.36e-21	0.0126
AUC	0.99 ± 0.00	0.85 ± 0.17	0.68 ± 0.08	0.78 ± 0.09	0.77 ± 0.28
MCC	1.00 ± 0.00	0.72 ± 0.32	0.41 ± 0.15	0.57 ± 0.17	0.57 ± 0.58

### 3.7 Systems-level translational mapping to clinical trials

Within an integrative systems framework, translational relevance is best assessed not as direct biomarker validation, but as the alignment between inferred molecular systems and existing pharmacological and interventional knowledge. To this end, we mapped each gene of the seven-gene system to curated drug–gene and drug–trial relationships using a merged, multi-source knowledge base (Table 4). This mapping is intended to contextualize the inferred 7-gene signature within the current therapeutic landscape rather than to claim clinical efficacy.

**TABLE 4:** Drugs, their gene targets, and number of associated clinical trials.

Drug name/Intervention	Targeted gene	Number of clinical trials
TOCILIZUMAB	IL6R	61
ASPIRIN	ECHS1	11
SARILUMAB	IL6R	10
IL-6–targeting interventions	IL6R	7
RIBAVIRIN	SOCS3	7
CLOPIDOGREL	ECHS1	2
SILDENAFIL	ARG1	2
THALIDOMIDE	IL6R	2
LEVILIMAB	IL6R	1
SIRUKUMAB	IL6R	1
VITAMIN E	PPP2CB	1

Five genes (*IL6R*, *ARG1*, *SOCS3*, *PPP2CB*, and *ECHS1*) exhibited documented links to approved or investigational compounds appearing in ongoing or completed clinical trials associated with COVID-19, Long COVID, or SARS-CoV-2. The convergence between algorithmically inferred molecular components and independently developed clinical trials provides an external, systems-level indication that the identified gene system is consistent with biologically relevant processes rather than dataset-specific artifacts.

#### 3.7.1 IL6R

Among the inferred components, *IL6R* shows the strongest alignment with existing clinical activity. More than 80 trials in the compiled dataset involve modulation of the *IL-6* signaling pathway, including interventions such as tocilizumab, sarilumab, siltuximab, sirukumab, and levilimab. In the compiled tables, *IL-6* appears as a drug-level label because it is used by clinical databases to index *IL-6*–targeted interventions, not because *IL-6* itself is administered therapeutically. These trials collectively reflect sustained clinical focus on cytokine signaling control across acute and post-acute disease stages, consistent with the central positioning of *IL6R* within the inferred immune–metabolic system.

#### 3.7.2 ARG1

Two trials in the dataset involve sildenafil-based interventions linked to *ARG1*-associated pathways. Although fewer in number, these studies point to therapeutic interest in vascular and metabolic modulation connected to arginine metabolism and immune regulation.

#### 3.7.3 SOCS3

Seven ribavirin-associated trials map to *SOCS3*, reflecting active investigation of antiviral and immune-modulatory strategies that intersect with JAK–STAT and cytokine feedback regulation. This alignment supports the role of *SOCS3* as a regulatory node within the broader system rather than as an isolated antiviral marker.

#### 3.7.4 PPP2CB

A single clinical study involving vitamin E is linked to *PPP2CB*, indicating limited but detectable translational exploration. While currently underrepresented in clinical trials, its recurrent selection across datasets suggests that it may act as a regulatory component whose therapeutic relevance has not yet been fully explored.

#### 3.7.5 ECHS1

Thirteen trials involving aspirin or clopidogrel are linked to *ECHS1*, connecting this mitochondrial-associated enzyme to interventions aimed at inflammatory and thrombotic processes. This correspondence supports the integration of metabolic and immune signaling components within the inferred system.

Taken together, more than 100 clinical trials are connected to compounds associated with the gene system identified in this study. This observation does not constitute clinical validation of individual targets, but it provides an external consistency check showing that the inferred system aligns with active therapeutic hypotheses pursued independently by the clinical research community. While drug–gene associations do not establish causal mechanisms, their convergence with the identified gene set provides independent support for the biological and translational relevance of the observed expression patterns. Within an integrative systems microbiology perspective, such alignment strengthens confidence that the algorithmically derived gene system is consistent with conserved biological patterns across datasets relevant across molecular, pharmacological, and interventional layers. The complete list of associated clinical trials is provided in the Annex (Supplementary Table S1).

## 4 Discussion and conclusion

This work addresses a central problem in integrative systems microbiology: how to infer stable, biologically interpretable molecular patterns from heterogeneous, small-scale omics datasets. Rather than treating Long COVID as an isolated disease entity, we use it as a representative case study to evaluate algorithmic strategies for multi-dataset and multi-knowledge-base integration. Our results show that emphasizing cross-cohort reproducibility and ensemble consensus enables the recovery of coherent biological systems that persist despite variation in tissue source, sequencing platform, and clinical context.

The MCC-REFS algorithm consistently identified a compact and interpretable multigene system across five independent transcriptomic cohorts. Previous work using REFS and MCC-REFS has demonstrated improved biomarker reproducibility across independent datasets and benchmarking platforms; the present study extends this framework to heterogeneous multi-cohort transcriptomic data spanning tissues and technologies. This stability is notable given the diversity of sample origins, including PBMCs, whole blood, plasma, and post-mortem tissue. The resulting seven-gene system (*PPP2CB*, *SOCS3*, *ARG1*, *IL6R*, *ECHS1*, *FZD2*, and *TRGV3/5*) reflects convergent immune-regulatory and metabolic processes rather than cohort-specific expression artifacts. Five of these genes (*PPP2CB*, *SOCS3*, *ARG1*, *IL6R*, and *ECHS1*) have been previously implicated in inflammatory signaling, immune feedback control, or mitochondrial function in the context of viral infection, supporting the biological plausibility of the inferred system.

**FIGURE 8:**
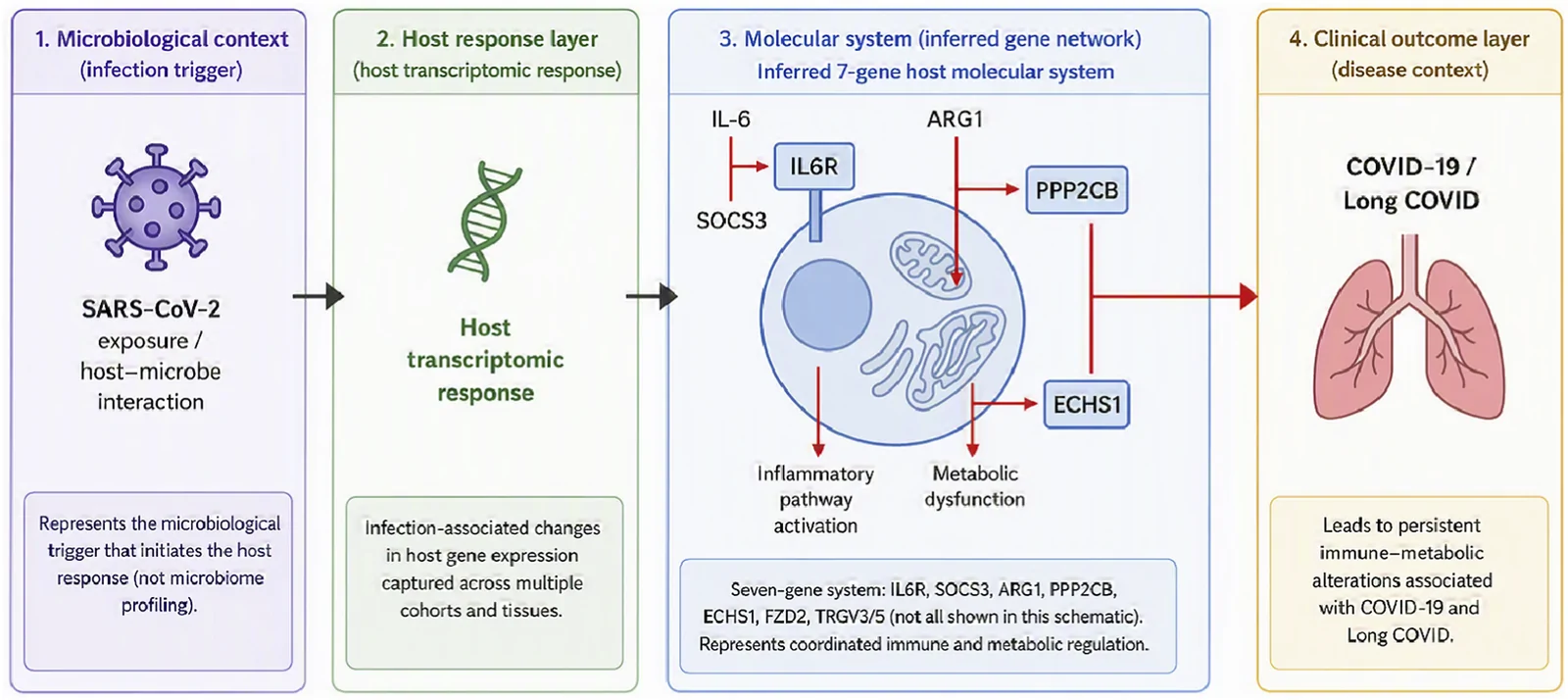
Integrative analysis of host transcriptomic responses to microbial infection across multiple datasets, tissues, and platforms. Note: This framework focuses on host-side molecular readouts of infection-associated processes; it does not represent direct microbiome analysis.

Importantly, the value of this result lies not in the novelty of individual genes, but in the reproducible emergence of a coordinated immune–metabolic expression pattern across datasets. Dysregulation of *IL6R*, *SOCS3*, and *ARG1* is consistent with sustained cytokine signaling and compensatory immune suppression, while reduced *ECHS1* expression points to altered mitochondrial fatty acid oxidation, a feature reported in post-viral fatigue and inflammatory syndromes (Yin et al., 2023; Fu et al., 2025). Together, these components suggest a linked pattern between inflammatory signaling and metabolic processes, rather than a set of independent biomarkers (Figure 8).

From a systems perspective, the generalization of this multigene structure across datasets is more informative than univariate significance within any single cohort. While several individual genes do not retain statistical significance after multiple-testing correction in all datasets, multivariate testing reveals strong joint effects, as reflected by consistent Wilks’ Lambda values across cohorts (Table 3). This behavior is characteristic of complex immune systems, where disease states are defined by coordinated perturbations rather than dominant single-gene effects. The results therefore reinforce the importance of algorithmic approaches that preserve multivariate structure instead of prioritizing sparse, cohort-dependent markers.

The integration of pharmacological and interventional knowledge bases provides an additional systems-level validation layer. Several components of the inferred gene system are already targeted by existing interventions, including IL-6 pathway inhibitors and agents modulating immune activation and metabolism (Ely and Center, 2025; ClinicalTrials.gov, 2021). This convergence between algorithmic inference and ongoing clinical investigation supports the relevance of the identified system. At the same time, genes such as *PPP2CB* and *ECHS1*, which are not yet primary clinical targets, emerge as potentially important regulatory nodes, highlighting directions for future mechanistic and experimental work. The observation that multiple genes from the identified signature have already been explored as therapeutic targets suggests that the signature captures biologically relevant processes rather than a random collection of genes.

We explicitly note that two external datasets (GSE157103 and E-ENAD-46) represent acute or fatal COVID-19 rather than Long COVID. In particular, the E-ENAD-46 dataset, generated early in the pandemic, can be interpreted as a surrogate for severe disease. These datasets are not used to demonstrate disease specificity, but to evaluate cross-context generalization of the inferred gene system across related SARS-CoV-2 conditions. The observed signal may therefore reflect shared inflammatory and immune–metabolic responses across disease stages rather than processes exclusive to Long COVID.

Several limitations should be considered. Sample sizes remain modest, and bulk transcriptomic data do not capture differences between individual cell types, which may contribute to residual variability (Yu et al., 2024). Although ensemble learning and cross-dataset validation help reduce batch effects, they do not fully eliminate them. In addition, experimental validation at the protein and functional levels is needed to confirm the role of individual components. However, the present study is designed as an algorithmic discovery and integration framework, focusing on identifying stable and reproducible transcriptomic patterns across heterogeneous datasets rather than providing direct experimental confirmation of underlying biological processes.

The consistency of feature selection further supports the robustness of the results, with some genes (such as SOCS3 and ARG1) consistently selected across resampling runs, while others showed more variable selection. To improve interpretation, we linked the genes to known drug targets and clinical studies. Because the gene set is small, standard pathway or network analyses are less reliable and may produce unstable results. Instead, we focus on a simple systems-level interpretation showing how these genes relate to immune and metabolic processes, while noting that future work could explore larger datasets or additional omics layers for more detailed pathway and network analyses.

In conclusion, this study demonstrates how algorithmic emphasis on stability, ensemble consensus, and multi-dataset integration supports biological interpretation in systems microbiology. The microbiology connection of this work lies in its focus on host-response patterns arising after microbial infection. The inferred gene system should therefore be interpreted as a host transcriptomic signature associated with SARS-CoV-2-related immune–metabolic states, rather than as a direct microbial or microbiome-derived signature. MCC-REFS allows the inference of reproducible immune–metabolic gene systems that persist across heterogeneous datasets and align with external pharmacological and clinical knowledge. While Long COVID serves as the motivating application, the proposed framework is broadly applicable to complex host–microbe and immunometabolic conditions where data heterogeneity and limited sample sizes challenge traditional analytical approaches. By shifting emphasis from single-cohort biomarker identification to cross-context algorithmic inference, this work provides a general framework for mechanistic integration in integrative systems microbiology. Because this study is based on observational transcriptomic data, the findings should be interpreted as associations rather than direct evidence of causal or regulatory mechanisms.

## Data Availability

The datasets analyzed in this study are publicly available from established repositories. Transcriptomic datasets GSE275334, GSE270045, GSE226260, and GSE157103 were obtained from the NCBI Gene Expression Omnibus (GEO) repository and are accessible at https://www.ncbi.nlm.nih.gov/geo/query/acc.cgi?acc%3cGSE275334, https://www.ncbi.nlm.nih.gov/geo/query/acc.cgi?acc=GSE270045, https://www.ncbi.nlm.nih.gov/geo/query/acc.cgi?acc=GSE226260, and https://www.ncbi.nlm.nih.gov/geo/query/acc.cgi?acc=GSE157103, respectively. Post-mortem tissue RNA-seq data were obtained from the EMBL-EBI Expression Atlas under accession number E-ENAD-46 and are available at https://www.ebi.ac.uk/gxa/experiments/E-ENAD-46. No new data were generated for this study. The code is available in the following repository: https://github.com/steppenwolf0/longCOVIDMCCREFS.

## Footnotes

1
https://scikit-learn.org/stable/modules/generated/sklearn.model_selection.StratifiedKFold.html

2
https://www.ncbi.nlm.nih.gov/geo/query/acc.cgi?acc=GSE275334

3
https://www.ncbi.nlm.nih.gov/geo/query/acc.cgi?acc=GSE270045

4
https://www.ncbi.nlm.nih.gov/geo/query/acc.cgi?acc=GSE226260

5
https://www.ncbi.nlm.nih.gov/geo/query/acc.cgi?acc=GSE157103

6
https://www.ebi.ac.uk/gxa/experiments/E-ENAD-46/Downloads
