# Supplementary material for: A systems microbiology framework for reproducible multi-dataset omics integration with application to long COVID
Source: Front Syst Biol. 2026 Sep 3;6:1873899. doi: 10.3389/fsysb.2026.1873899 (PMC13581729; doi:10.3389/fsysb.2026.1873899)
Supplement: Supplementary file 1 [file DataSheet1.pdf]

Supplementary Material

1 SUPPLEMENTARY DATA

Algorithm 1 MCC–REFS with External MLP Validation and Target–Disease Mapping

Require: Expression matrix $\mathbf{X} \in \mathbb{R}^{n \times p}$, labels $\mathbf{y} \in \{0, 1\}^n$, outer folds K_{out}, inner folds K_{in}, resamples R for stability, candidate feature counts $\mathcal{K}$ (optional if k is selected automatically), discovery cohort $\mathcal{D}$, external cohorts $\{\mathcal{E}_j\}$

Ensure: Final signature $S^\star$, stability indices, external AUCs, DrugBank/OpenTargets evidence

Outer CV for unbiased estimation: Split $(\mathbf{X}, \mathbf{y})$ into K_{out} stratified folds $\{\mathcal{T}_o, \mathcal{V}_o\}_{o=1}^{K_{\text{out}}}$.
for $o = 1$ to K_{out} **do**
  Inner CV feature selection (MCC–REFS) on $\mathcal{T}_o$:
    Partition $\mathcal{T}_o$ into K_{in} folds.
    Loop over resamples $r = 1, \dots, R$:
      Train an *ensemble* of base learners (8 diverse classifiers) and score features with *Matthews Correlation Coefficient* (MCC).
      Recursively eliminate low-ranked features; record ranked lists across inner folds.
      Determine k_r (either automatic selection that maximizes inner MCC or from $\mathcal{K}$).
    End loop; aggregate ranks/frequencies across R resamples to obtain the inner-selected subset S_o of size $k^\star$.
  Stability quantification: compute pairwise Jaccard and Nogueira indices across the R resamples.
  External model training (MLP): Fit an MLP classifier *not used in the MCC–REFS ensemble* on $\mathcal{T}_o$ restricted to S_o and evaluate on $\mathcal{V}_o$ (store AUC/ROC and other metrics).
end for
Aggregate performance: report mean±sd AUC over outer folds.
Signature consolidation $S^\star$: Select final genes by frequency (e.g., appearance threshold across outer folds) or by refitting MCC–REFS on the full discovery set $\mathcal{D}$ to the chosen $k^\star$.
External cohort validation:
for each external cohort $\mathcal{E}_j$ **do**
  Project $\mathcal{E}_j$ to $S^\star$, train MLP within cohort via stratified CV, report AUC/ROC.
end for
Translational validation (targets and drugs):
  Open Targets: For each gene $g \in S^\star$, query target–disease evidence (COVID-19 / Long COVID), record evidence types/scores and associated trials.
  DrugBank: For each gene/protein $g \in S^\star$, retrieve drug–target links; tag drug status (approved/investigational) and mechanisms.
  DGIdb: For each gene/protein $g \in S^\star$, retrieve drug–target links; tag drug status (approved/investigational) and mechanisms.
Output $S^\star$, stability indices (Jaccard, Nogueira), all AUCs, and Drug Targets summaries.

Table S1: Full list of found Clinical trials, with gene target.

Drug Name	NCT ID	phase_status	Target Gene
SILDENAFIL	NCT04489446	PHASE1, PHASE2 COMPLETED	ARG1
SILDENAFIL	NCT04304313	PHASE3 UNKNOWN	ARG1

Table S1: Full list of found Clinical trials, with gene target.

Drug Name	NCT ID	phase_status	Target Gene
ASPIRIN	NCT02735707	PHASE3 RECRUITING	ECHS1
ASPIRIN	NCT04333407	NA TERMINATED	ECHS1
ASPIRIN	NCT04363840	PHASE2 WITHDRAWN	ECHS1
ASPIRIN	NCT04365309	PHASE2, PHASE3 UNKNOWN	ECHS1
ASPIRIN	NCT04381936	PHASE3 RECRUITING	ECHS1
ASPIRIN	NCT04410328	PHASE3 COMPLETED	ECHS1
ASPIRIN	NCT04425863	NA COMPLETED	ECHS1
ASPIRIN	NCT04498273	PHASE3 TERMINATED	ECHS1
ASPIRIN	NCT05797597	PHASE3 UNKNOWN	ECHS1
ASPIRIN	NCT04324463	PHASE3 COMPLETED	ECHS1
ASPIRIN	NCT04343001	PHASE3 WITHDRAWN	ECHS1
CLOPIDOGREL	NCT04368377	PHASE2 COMPLETED	ECHS1
CLOPIDOGREL	NCT04409834	PHASE4 COMPLETED	ECHS1
IL-6	NCT04517630	NA COMPLETED	IL6R
IL-6	NCT05421208	NA RECRUITING	IL6R
IL-6	NCT06027255	NA RECRUITING	IL6R
IL-6	NCT04486521	NA UNKNOWN	IL6R
IL-6	NCT05218369	NA UNKNOWN	IL6R
IL-6	NCT05330845	NA TERMINATED	IL6R
IL-6	NCT05638932	NA COMPLETED	IL6R
LEVILIMAB	NCT04397562	PHASE3 COMPLETED	IL6R
SARILUMAB	NCT04315298	PHASE2, PHASE3 COMPLETED	IL6R
SARILUMAB	NCT04341870	PHASE2, PHASE3 SUSPENDED	IL6R
SARILUMAB	NCT04357808	PHASE2 COMPLETED	IL6R
SARILUMAB	NCT04359901	PHASE2 COMPLETED	IL6R
SARILUMAB	NCT07196306	PHASE2 WITHDRAWN	IL6R
SARILUMAB	NCT04324073	PHASE2, PHASE3 COMPLETED	IL6R
SARILUMAB	NCT04327388	PHASE3 COMPLETED	IL6R
SARILUMAB	NCT04357860	PHASE2 COMPLETED	IL6R
SARILUMAB	NCT04386239	EARLY_PHASE1 WITHDRAWN	IL6R
SARILUMAB	NCT04661527	PHASE2 UNKNOWN	IL6R
SIRUKUMAB	NCT04380961	PHASE2 COMPLETED	IL6R
THALIDOMIDE	NCT04273529	PHASE2 UNKNOWN	IL6R
THALIDOMIDE	NCT04273581	PHASE2 UNKNOWN	IL6R
TOCILIZUMAB	NCT04310228	NA UNKNOWN	IL6R
TOCILIZUMAB	NCT04315480	PHASE2 UNKNOWN	IL6R
TOCILIZUMAB	NCT04321993	PHASE2 UNKNOWN	IL6R
TOCILIZUMAB	NCT04332094	PHASE2 UNKNOWN	IL6R
TOCILIZUMAB	NCT04335071	PHASE2 TERMINATED	IL6R
TOCILIZUMAB	NCT04339712	PHASE2 COMPLETED	IL6R
TOCILIZUMAB	NCT04349410	PHASE2, PHASE3 COMPLETED	IL6R
TOCILIZUMAB	NCT04361552	PHASE3 WITHDRAWN	IL6R

Table S1: Full list of found Clinical trials, with gene target.

Drug Name	NCT ID	phase_status	Target Gene
TOCILIZUMAB	NCT04363853	PHASE2 ACTIVE_NOT_RECRUITING	IL6R
TOCILIZUMAB	NCT04370834	PHASE2 TERMINATED	IL6R
TOCILIZUMAB	NCT04377750	PHASE4 UNKNOWN	IL6R
TOCILIZUMAB	NCT04380818	NA UNKNOWN	IL6R
TOCILIZUMAB	NCT04403685	PHASE3 TERMINATED	IL6R
TOCILIZUMAB	NCT04412291	PHASE2 UNKNOWN	IL6R
TOCILIZUMAB	NCT04423042	PHASE3 UNKNOWN	IL6R
TOCILIZUMAB	NCT04678739	PHASE3 COMPLETED	IL6R
TOCILIZUMAB	NCT04693026	PHASE3 UNKNOWN	IL6R
TOCILIZUMAB	NCT04730323	PHASE4 COMPLETED	IL6R
TOCILIZUMAB	NCT04924829	NA UNKNOWN	IL6R
TOCILIZUMAB	NCT05133635	PHASE4 WITHDRAWN	IL6R
TOCILIZUMAB	NCT05925140	PHASE1 UNKNOWN	IL6R
TOCILIZUMAB	NCT04306705	NA UNKNOWN	IL6R
TOCILIZUMAB	NCT04317092	PHASE2 UNKNOWN	IL6R
TOCILIZUMAB	NCT04320615	PHASE3 COMPLETED	IL6R
TOCILIZUMAB	NCT04330638	PHASE3 COMPLETED	IL6R
TOCILIZUMAB	NCT04331795	PHASE2 COMPLETED	IL6R
TOCILIZUMAB	NCT04331808	PHASE2 COMPLETED	IL6R
TOCILIZUMAB	NCT04335305	PHASE2 TERMINATED	IL6R
TOCILIZUMAB	NCT04345445	PHASE3 UNKNOWN	IL6R
TOCILIZUMAB	NCT04346355	PHASE2 TERMINATED	IL6R
TOCILIZUMAB	NCT04347031	PHASE2, PHASE3 COMPLETED	IL6R
TOCILIZUMAB	NCT04356937	PHASE3 COMPLETED	IL6R
TOCILIZUMAB	NCT04359667	NA UNKNOWN	IL6R
TOCILIZUMAB	NCT04361032	PHASE3 UNKNOWN	IL6R
TOCILIZUMAB	NCT04372186	PHASE3 COMPLETED	IL6R
TOCILIZUMAB	NCT04377503	PHASE2 TERMINATED	IL6R
TOCILIZUMAB	NCT04377659	PHASE2 TERMINATED	IL6R
TOCILIZUMAB	NCT04394182	NA SUSPENDED	IL6R
TOCILIZUMAB	NCT04409262	PHASE3 COMPLETED	IL6R
TOCILIZUMAB	NCT04412772	PHASE3 TERMINATED	IL6R
TOCILIZUMAB	NCT04424056	PHASE3 UNKNOWN	IL6R
TOCILIZUMAB	NCT04435717	PHASE2 TERMINATED	IL6R
TOCILIZUMAB	NCT04445272	PHASE2 COMPLETED	IL6R
TOCILIZUMAB	NCT04476979	PHASE2 COMPLETED	IL6R
TOCILIZUMAB	NCT04479358	PHASE2 TERMINATED	IL6R
TOCILIZUMAB	NCT04492501	NA COMPLETED	IL6R
TOCILIZUMAB	NCT04519385	NA COMPLETED	IL6R
TOCILIZUMAB	NCT04560205	PHASE1 UNKNOWN	IL6R
TOCILIZUMAB	NCT04600141	PHASE3 COMPLETED	IL6R
TOCILIZUMAB	NCT04690920	NA COMPLETED	IL6R

Table S1: Full list of found Clinical trials, with gene target.

Drug Name	NCT ID	phase_status	Target Gene
TOCILIZUMAB	NCT04734678	NA COMPLETED	IL6R
TOCILIZUMAB	NCT04779047	PHASE4 UNKNOWN	IL6R
TOCILIZUMAB	NCT04871854	PHASE2 COMPLETED	IL6R
TOCILIZUMAB	NCT04873141	NA COMPLETED	IL6R
TOCILIZUMAB	NCT04893031	NA COMPLETED	IL6R
TOCILIZUMAB	NCT05035589	NA COMPLETED	IL6R
TOCILIZUMAB	NCT05082714	NA COMPLETED	IL6R
TOCILIZUMAB	NCT05164133	PHASE1 COMPLETED	IL6R
TOCILIZUMAB	NCT05279391	NA UNKNOWN	IL6R
TOCILIZUMAB	NCT05302947	NA COMPLETED	IL6R
TOCILIZUMAB	NCT06233357	NA COMPLETED	IL6R
VITAMIN E	NCT04570254	NA COMPLETED	PPP2CB
RIBAVIRIN	NCT04276688	PHASE2 COMPLETED	SOCS3
RIBAVIRIN	NCT04392427	PHASE3 UNKNOWN	SOCS3
RIBAVIRIN	NCT04828564	PHASE2, PHASE3 UNKNOWN	SOCS3
RIBAVIRIN	NCT04494399	PHASE2 UNKNOWN	SOCS3
RIBAVIRIN	NCT04605588	PHASE2 TERMINATED	SOCS3
RIBAVIRIN	NCT04664010	NA COMPLETED	SOCS3
RIBAVIRIN	NCT04959786	PHASE2, PHASE3 UNKNOWN	SOCS3
